# Type 2 diabetes remission trajectories and variation in risk of diabetes complications: A population-based cohort study

**DOI:** 10.1371/journal.pone.0290791

**Published:** 2023-08-29

**Authors:** Hajira Dambha-Miller, Hilda O. Hounkpatin, Beth Stuart, Andrew Farmer, Simon Griffin

**Affiliations:** 1 Primary Care Research Centre, School of Primary Care Population Sciences and Medical Education, University of Southampton, Southampton, England; 2 Nuffield Department of Primary Care Health Sciences, University of Oxford, Oxford, England; 3 Department of Public Health and Primary Care, School of Clinical Medicine, University of Cambridge, Cambridge, England; 4 MRC Epidemiology Unit, School of Clinical Medicine, University of Cambridge, Cambridge, England; University of Botswana School of Medicine, BOTSWANA

## Abstract

Biochemical remission of type 2 diabetes is achievable through dietary changes, physical activity and subsequent weight loss. We aim to identify distinct diabetes remission trajectories in a large population-based cohort over seven-years follow-up and to examine associations between remission trajectories and diabetes complications. Group-based trajectory modelling examined longitudinal patterns of HbA_1c_ level (adjusting for remission status) over time. Multivariable Cox models quantified the association between each remission trajectory and microvascular complications, macrovascular complications, cardiovascular (CVD) events and all-cause mortality. Four groups were assigned. Group 1 (8,112 [13.5%]; achieving HbA_1c_ <48 mmol/mol (6.5%) followed by increasing HbA_1c_ levels); Group 2 (6,369 [10.6%]; decreasing HbA_1c_ levels >48 mmol/mol (6.5%)); Group 3 (36,557 [60.6%]; stable high HbA_1c_ levels); Group 4 (9,249 [15.3%]; stable low HbA_1c_ levels (<48mmol/mol or <6.5%)). Compared to Group 3, Groups 1 and 4 had lower risk of microvascular complications (aHRs (95% CI): 0.65 (0.61–0.70), p-value <0.001;0.59 (0.55–0.64) p-value<0.001, respectively)), macrovascular complications (aHRs (95% CI): 0.83 (0.75–0.92), p-value<0.001; 0.66 (0.61–0.71), p-value<0.001) and CVD events (aHRs (95% CI): 0.74(0.67–0.83), p-value<0.001; 0.67(0.61–0.73), p-vlaue<0.001). Risk of CVD outcomes were similar for Groups 2 and 3. Compared to Group 3, Group 1 (aHR: 0.82(95% CI: 0.76–0.89)) had lower risk of mortality, but Group 4 had higher risk of mortality (aHR: 1.11(95% CI: 1.03–1.19)). Risk of CVD outcomes vary by pattern of remission over time, with lowest risk for those in remission longer. People who achieve remission, even for shorter periods of time, continue to benefit from this lower exposure to hyperglycaemia, which may, in turn, lower the risk of CVD outcomes including mortality.

## Introduction

Type 2 diabetes affects 463 million adults globally corresponding to 6.28% of the world’s population and total diabetes-related health expenditure is estimated to be over £570 billion [1]. This substantial economic burden is in part related to associated cardiovascular disease (CVD). People with type 2 diabetes compared to those without are more likely to have CVD including peripheral arterial disease, ischaemic stroke, stable angina, heart failure, and non-fatal myocardial infarction. Intensive multifactorial management is effective at reducing these complications, and recent evidence demonstrates that biochemical remission of the disease is achievable through dietary changes, physical activity and subsequent weight loss [2,3]. Remission is defined as a level of glycaemia below the diagnostic threshold (HbA_1c_ < 6.5% or 48 mmol/mol) in the absence of medication or bariatric surgery. We have previously demonstrated that ≥ 10% weight loss achieved early after diagnosis is strongly associated with remission ((RR 2.43 (95% CI 1.78 to 3.31, p<0.01)) [4]. Remission is often temporary and over the course of type 2 diabetes, individuals will move between states of remission and relapse. To date, these longitudinal patterns of remission have not been described in large population-based cohorts.

It is plausible that since remission is defined by HbA_1c_ level, previous studies on the association between glycaemia and CVD outcomes might be comparable. Observational studies consistently demonstrate a positive association between glycaemia and CVD [5], whereas evaluations of interventions to lower glucose report heterogeneous findings [6–8]. The Look Ahead trial of an intensive behavioural intervention was terminated due to futility in relation to the CVD endpoint [9]. The results of trials of pharmacological interventions to lower glucose have varied according to the drugs (or combination of drugs) undergoing evaluation, the speed and extent of reduction of glucose levels, participants’ existing CVD risk and their point in the disease trajectory at baseline [6–8]. Extrapolation of these findings to characterise the impact of remission on the development of CVD outcomes is therefore challenging. To our knowledge, one study has examined the impact of remission on long-term CVD outcomes, with earlier studies focusing on short-term CVD outcomes [4,10–12]. This study reported that remission was associated with lower risk of microvascular complications, macrovascular complications, and CVD events [12]. However, it is unclear whether this risk varies by different patterns of remission over time. This knowledge could inform clinical and policy initiatives which have recently been promoting biochemical remission as a target for management of type 2 diabetes. Accordingly, in this study we describe longitudinal patterns of remission in a large population-based cohort and model these into distinct groups over seven-year follow-up. We then examine risk of CVD outcomes, and all-cause mortality overall and by pattern of remission.

## Materials and methods

### Design

A retrospective cohort study.

### Data source

The Electronic Care and Health Information Analytics (CHIA) database is a pseudo-anonymised live electronic database with routinely collected primary care data for approximately 1.5 million people from 150 primary care practices across Hampshire and the Isle of Wight (Southern England, UK) with linked clinical and biochemistry data from local hospitals.

### Population

We identified a cohort of people with type 2 diabetes using the Quality and Outcomes Framework (QOF) Read code diagnosis. QOF coding is used for NHS administration and financial purposes with high levels of accuracy/completeness [13]. From 120,000 people coded with type 2 diabetes by this criteria on the 1^st^ January 2013, we included 60,287 in our cohort who also had linked and continuous records for seven years until 1^st^ April 2020 (or death) and for whom remission status could be assessed.

### Exposure

Remission was defined as having two HbA_1c_ level < 48 mmol/mol (6.5%) measurements separated over a period of at least six months in the absence of diabetes medications or bariatric surgery [14]. Remission status was assessed for people with HbA_1c_ data for at least two follow-up measurements (i.e., those surviving for at least the first 12 months of follow up).

### Outcomes

- Macrovascular complication as a composite of stroke, myocardial infarct (MI) coronary heart disease (CHD), peripheral arterial disease (PAD), or amputation
- Microvascular complications as a composite of peripheral neuropathy, retinopathy, and nephropathy
- CVD events as a composite of MI, amputation, and stroke
- All-cause mortality

We used QoF definitions for peripheral neuropathy, retinopathy, and nephropathy and these were captured using read codes from the primary care record. There was complete data on each outcome measure as a result of the linked data.

### Covariates

#### Sociodemographic characteristics

Baseline data were extracted on age, sex, ethnicity (White, Black, Asian, Mixed and other) and socioeconomic status. This was defined using the 2019 Index of Multiple Deprivation (IMD) quintiles which is a small-area measure of socioeconomic status, ranked nationally and comprises seven domains: income, employment, education/skills/training, health and disability, crime, barriers to housing and services, and living environment) were available. IMD 1 represents the most deprived and IMD 5 represents the least deprived groups.

#### Clinical variables

Baseline comorbidities were defined from diagnostic codes from existing QOF conditions including coronary heart disease, chronic kidney disease, chronic obstructive pulmonary disease (COPD), asthma, cancer, dementia, atrial fibrillation, epilepsy, heart failure, stroke, peripheral vascular disease, hypertension, osteoporosis, osteoarthritis, and depression. Frailty was defined using the electronic frailty Index score. Latest smoking status was extracted at the start of the study (1^st^ January 2013). Weight, body mass index (BMI), systolic and diastolic blood pressure and biochemistry measures (including HbA_1c_ total cholesterol, HDL-cholesterol and eGFR) were taken between January 2013 and April 2020 in six-month intervals, where available. For baseline, we used measures recorded between 1^st^ January 2013-1^st^ April 2013.

#### Medication

Prescribed repeat medication data were extracted from the electronic record at 6-month intervals for the duration of the study period. We used the prescriptions between 1^st^ January 2013-1^st^ April 2013 as the baseline.

### Ethics statement

CHIA is an anonymous National Health Service database and all individuals have consented for collection of their medical records for inclusion in the database (written consent). Ethical and governance approval for this study was obtained from the University of Southampton (ERGO 56127), and Care and Health Information Exchange Information Governance Group (CHIE IGG). All data were fully anonymised prior to the research team gaining access to the data.

### Statistical analysis

We summarised baseline characteristics of the whole cohort. There were missing data on ethnicity (49%) and IMD (0.9%). Ethnicity is frequently missing from routinely collected primary care records and we assigned missing data into the white category in keeping with the local population and previous studies [15]. For weight and HbA_1c_ data that were missing (n = 29678 (49.2%) and n = 30002 (49.8%)), we assumed missing at random and imputed these in a model that included the following non-missing variables; age, sex, diabetes duration, total number of comorbidities at baseline, practice ID, and outcome variables. Data were multiply imputed using Markov Chain Monte Carlo using STATA SE 16.0. We used 10 cycles of imputation. Separate similar imputation models were applied for the remaining biochemistry data. All imputed data after patient death were recoded as missing. With a complete dataset, we summarised participant characteristics stratified by remission status.

We then modelled trajectories of HbA_1c_ level (as a binary measure indicating 48 mmol/mol (6.5%) and above or below 48 mmol/mol) over time and adjusting for remission status at each time point using group-based trajectory modelling in STATA (program developed by Jones and Nagin and based on imputed HbA_1c_ data) [16]. Group-based trajectory models (GBTMs) are mixture models that assume a population is composed of a mixture of distinct subgroups of people who have similar developmental trajectories. A series of unadjusted GBTMs were applied to fit 1 through to 6 group models. The shape of the trajectory was determined by first fitting the trajectory as a cubic function and then reducing the function (to quadratic, slope, or intercept only) if higher polynomials were not statistically significant. The number of trajectories in the model was increased by one and the steps were repeated. Participants are assigned to the group they have highest probability of belonging. We considered participants as belonging to a group if the classification probability was >0.80. The best-fitting model was selected based on 3 criteria: (1) the Bayesian Information Criterion (BIC) (where a lower BIC indicates better fit) (2) the odds of correct classification into each group and (3) the average posterior probabilities of group membership, as a measure of classification quality (>0.80 or greater in all group) [16].The best-fitting model was fitted to each imputed dataset and the classification probabilities from each dataset were saved and averaged to determine group membership. We then used descriptive statistics to compare baseline sociodemographic and clinical characteristics for each remission group. Model F statistics were used to test differences in variables across the groups. Multinomial models (unadjusted and adjusted for age, sex, ethnicity, IMD, baseline weight, diabetes duration, number of comorbidities and clustering within practices) were used to examine the association between weight change categories (no change or weight gain, weight loss (≤ 2.5–5%), (≤5–10%) and (≥10%) from baseline weight) and group membership. We examine associations between weight change categories and remission groups as previous studies have found an association with overall remission [5,17].

We fitted multivariable-adjusted Cox proportional hazards models to quantify the association between remission at any point during the follow-up for the whole cohort and the incidence of i) macrovascular complications ii) microvascular complications, iii) CVD events, and iv) all-cause mortality). We then constructed the same models with each distinct remission group. People with the event of interest before the start of study were excluded from the respective analysis. Quarter of death rather than exact date of death was available in the database therefore, the mid-point of the quarter of death was used as the date of death in the time to event analyses. For participants with multiple outcome events, we used the time the first event occurred in our time to event analyses. Multivariable models were adjusted based on a priori reasoning for age, sex, ethnicity, IMD, baseline weight, diabetes duration, number of co-morbidities and clustering within practices. Finally, we ran a sensitivity analysis to test the robustness of our imputation methods by re-running the cox models and including only those with non-missing (non-imputed) data. A p-value of <0.05 was considered as statistical significance in all analyses.

## Results

### Baseline population characteristics

The cohort included 60,287 people with type 2 diabetes with a mean duration of follow-up of 6.9 years. 7,312 (12.1%) people died during follow up. The mean age of the cohort was 64.6 years, most were male (n = 34,408, 57.1%), white (n = 58,148, 96.5%) and with a mean (SD) duration of diabetes of 8.1 (6.8) years. Baseline characteristics are summarised in Table 1. During the 7-year follow-up period, 11,491 (19.1%) people achieved remission at some point for at least a 6-month period. People who achieved remission compared to those who did not were older (p<0.001), more likely to be female (p<0.001), non-smokers (p<0.001), from a less deprived area (p<0.001) and with a lower baseline weight (p<0.001). Those not included in our study cohort (i.e., those diagnosed with diabetes after 1^st^ January 2013 or with less than seven years continuous data) were younger [mean (SD) 58.1 (14.2)], had shorter diabetes duration [mean (SD) = 5.0 (6.7)] and fewer comorbidities at baseline [mean (SD) 0.9 (1.1)], and slightly higher weight at baseline ([mean (SD) 94.2 (0.2)].

**Table 1:** Baseline characteristics of the type 2 diabetes cohort within the CHIA database stratified by remission status^¥^.

		All (n = 60287)		Remission^¥^ (n = 11335)		Non-remission^¥^ (n = 48,607)
**Sociodemographic**						
Age, years*		64.6 (12.0)		66.3 (11.9)		64.1 (12.0)
Male gender, n (%)		34408 (57.1)		5992 (52.9)		28224 (58.1)
Ethnicity, n (%)						
White		58148 (96.5)		11047 (97.5)		46767 (96.2)
Black		217 (0.4)		35 (0.3)		181 (0.4)
Asian		1514 (2.5)		190 (1.7)		1317 (2.7)
Mixed/Other		408 (0.7)		63 (0.6)		342 (0.7)
Socioeconomic Status, n (%)						
Index of Multiple Deprivation quintile 1		7576 (12.6)		1173 (10.3)		6363 (13.1)
(most deprived)						
Index of Multiple Deprivation quintile 2		12137 (20.1)		2280 (20.1)		9788 (20.1)
Index of Multiple Deprivation quintile 3		11457 (19.0)		2018 (17.8)		9375 (19.3)
Index of Multiple Deprivation quintile 4		13028 (21.6)		2554 (22.5)		10398 (21.4)
Index of Multiple Deprivation quintile 5 (least deprived)		16089 (26.7)		3310 (29.2)		12683 (26.1)
Clinical						
Diabetes duration, years (n = 60138)	60138	8.1 (6.8)	11324	5.9 (5.3)	48469	8.7 (7.0)
Frailty Index (n = 60244)		0.2 (0.1)	11316	0.2 (0.1)	48583	0.2 (0.1)
Total number baseline comorbidities n (%)		1.3 (1.2)		1.4 (1.2)		1.3 (1.2)
Hypertension, n (%)		30868 (51.2)		5968 (52.7)		24721 (50.9)
Stroke n (%)		2584 (4.3)		541 (4.8)		2027 (4.2)
Myocardial Infarction n (%)		4208 (7.0)		702 (6.2)		3483 (7.2)
Amputation n (%)		648 (1.1)		85 (0.7)		560 (1.2)
Current smoker, n (%)		6559 (10.9)		1177 (10.4)		5345 (11.0)
Weight, kg*		90.8 (20.7)		88.7 (20.4)		91.4 (20.7)
BMI, kg/m^2^*		31.5 (6.3)		30.9 (6.3)		31.7 (6.3)
Systolic blood pressure, mmHg*		136.1 (15.4)		136.2 (15.5)		136.1 (15.4)
Diastolic blood pressure, mmHg*		77.2 (9.4)		77.2 (9.4)		77.2 (9.4)
Total cholesterol, mmol/l*		4.6 (1.2)		4.7 (1.2)		4.5 (1.2)
HDL cholesterol, mmol/l*		1.2 (0.4)		1.3 (0.4)		1.2 (0.3)
HbA_1c_ level, mmol/mol*		60.1 (20.4)		59.3 (19.7)		60.2 (20.6)
eGFR		73.1 (17.1)		72.6 (17.2)		73.2 (17.0)
Total number of medications prescribed^#^		3.9 (2.4)		3.3 (2.4)		4.1 (2.4)
Anti-hypertensive medication, n (%)		32509 (53.9)		6071 (53.6)		26253 (54.0)
Lipid-lowering medication, n (%)		40992 (68.0)		6903 (60.9)		33862 (69.7)
Hypoglycaemic medication, n(%)		41085 (68.1)		4087(36.1)		36799 (75.7)

*Mean (SD). Remission was defined as having two HbA_1c_ < 6.5% (48mmol/mol) readings separated by at least a period of 6 months and no oral hypoglycaemic medication and no history of bariatric surgery

^¥^Estimation sample varies across imputations; minimum number of observations reported. Baseline biochemistry data was defined as the mean of any measurements taken between 1^st^ January 2013 and 31^st^ March 2013) ^#^Medication was defined as being prescribed during the first 6 months of the follow-up year (i.e., Jan-Jul 2013).

### Characteristics of groups by remission trajectory

**Fig 1:**
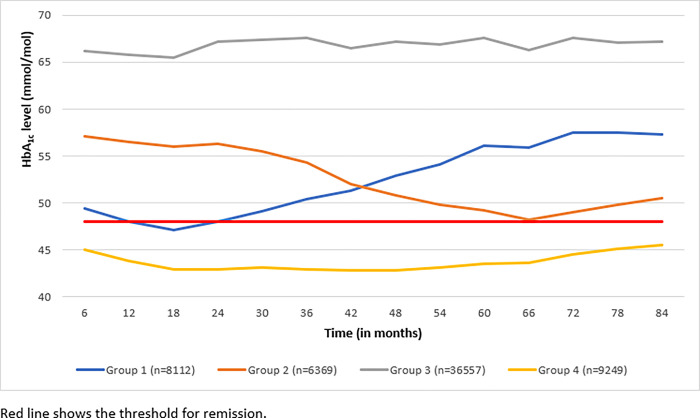
Mean HbA_1c_ level for each remission group over seven-year follow-up within the CHIA type 2 diabetes cohort (n = 60,287).

The best fitting model identified 4 groups with varying patterns of HbA_1c_ level and remission: Group 1 (8,112 [13.5%]; achieving HbA_1c_ <48 mmol/mol (6.5%) followed by increasing HbA_1c_ levels); Group 2 (6,369 [10.6%]; decreasing HbA_1c_ levels); Group 3 (36,557 [60.6%]; stable high HbA_1c_ levels); Group 4 (9,249 [15.3%]; stable low HbA_1c_ levels (<48mmol/mol or <6.5%)). Fig 1 presents the mean HbA_1c_ levels overtime for each group.

The sociodemographic and clinical characteristics for each remission group are summarised in Table 2 below. There were statistically significant differences in characteristics across the four groups. Individuals in Group 2, who had decreasing HbA_1c_ levels, were mainly older and living in less deprived areas. Those in group 3 who had increasing HbA_1c_ levels were younger, more likely to be male, current smokers, and had a longer duration of diabetes. Those in Group 1 (who achieved HbA_1c_ levels <48mmol/mol (6.5%) but then had increased HbA_1c_ levels) were also younger but had shorter diabetes duration and fewer medications prescribed. Those who had low (<48 mmol/mol (6.5%)) HbA_1c_ levels throughout follow-up (Group 4) were likely to be older, living in less deprived areas, with the shortest duration of diabetes and a lower baseline weight (Table 2). Group 3 was selected as the reference group in regression models as this was the group with high HbA_1c_ levels throughout the study and therefore a useful comparator to estimate associations with the different remission trajectories. Multinomial regression models indicated that, compared to those who did not change weight or those that gained weight, patients who achieved weight loss of ≥10% at 18 months follow-up were more likely to be in group 4 compared to group 3 (unadjusted relative risk ratio [RRR] (95% CI): 1.13 (1.06–1.21); adjusted RRR (95% CI): 1.42 (1.31–1.54)) (Table 3). Patients who achieved weight loss of ≥10% at 18 months follow-up were less likely to be in Group 2 compared to Group 3 (unadjusted RRR (95% CI): 0.89 (0.81–0.98); adjusted RRR (95% CI): 0.89 (0.81–0.98). Weight loss of ≥10% at 18 months follow-up was associated with Group 1 (compared to Group 3) in adjusted models but not unadjusted models (Table 3). Weight change at 18 months follow-up was examined as this was the time point at which key differences in HbA_1c_ were noted in Fig 1.

**Table 2:** Baseline characteristics by remission group in the CHIA type 2 diabetes cohort (n = 60,287).

	Group 1 Achieving HbA_1c_ <48mmol/mol (6.5%) followed by increasing HbA_1c_ levels	Group 2 Decreasing HbA_1c_ levels (>48mmol/mol)	Group 3 Stable high HbA_1c_ levels	Group 4 Stable low HbA_1c_ levels	P-value^#^
N (%)	8112 (13.8)	6369 (10.7)	36557 (60.0)	9249 (15.5)	
**Sociodemographic**					
Age, years*	65.4 (11.6)	66.8 (11.1)	63.1 (12.1)	67.8 (11.3)	0.419
Male gender, n (%)	4598 (56.7)	3609 (56.7)	21302 (58.3)	4899 (53.0)	0.006
Ethnicity, n (%)
White	7772 (95.8)	6132 (96.3)	35239 (96.4)	9005 (97.4)	
Black	25 (0.3)	25 (0.4)	137 (0.4)	30 (0.3)	0.858
Asian	247 (3.0)	162 (2.5)	944 (2.6)	161 (1.7)	<0.001
Mixed/Other	68 (0.8)	50 (0.8)	237 (0.6)	53 (0.6)	0.012
Index of Multiple Deprivation, n (%)
quintile 1 (most deprived)	871 (10.8)	750 (11.8)	4979 (13.6)	976 (10.5)	0.003
quintile 2	1575 (19.4)	1203 (18.9)	7631 (20.9)	1727 (18.7)	
quintile 3	1487 (18.3)	1151 (18.1)	7146 (19.5)	1672 (18.1)	
quintile 4	1828 (22.5)	1413 (22.2)	7702 (21.1)	2086 (22.5)	
quintile 5 (least deprived)	2351 (29.0)	1852 (29.1)	9099 (24.9)	2788 (30.2)	
**Clinical**					
Diabetes duration, years	6.7 (6.2)	7.5 (6.1)	9.1 (7.2)	6.3 (5.6)	<0.001
Frailty Index	0.2 (0.1)	0.2 (0.1)	0.2 (0.1)	0.2 (0.1)	0.043
Total number baseline comorbidities n(%)	1.3 (1.2)	1.4 (1.2)	1.2 (1.2)	1.4 (1.3)	0.038
Hypertension, n (%)	4434 (54.7)	3573 (56.1)	177726 (48.6)	5089 (55.0)	<0.001
Stroke n (%)	358 (4.4)	297 (4.7)	1448 (4.0)	481 (5.2)	0.353
Myocardial Infarction n (%)	518 (6.4)	457 (7.2)	2638 (7.2)	595 (6.4)	0.537
Amputation n (%)	67 (0.8)	79 (1.2)	428 (1.2)	74 (0.8)	0.840
Current smoker, n (%)	832 (10.3)	672 (10.6)	4117 (11.3)	938 (10.1)	0.416
Weight, kg*	89.2 (20.3)	90.5 (20.7)	92.1 (20.7)	87.8 (20.3)	0.954
BMI, kg/m^2^*	31.3 (6.3)	32.0 (6.4)	31.7 (6.3)	30.7 (6.3)	<0.001
Systolic blood pressure, mmHg*	136.0 (15.2)	136.9 (15.4)	136.1 (15.4)	135.7 (15.5)	0.128
Diastolic blood pressure, mmHg*	77.1 (9.4)	77.0 (9.4)	77.5 (9.4)	76.4 (9.3)	0.044
Total cholesterol, mmol/l*	4.6 (1.2)	4.5 (1.2)	4.6 (1.2)	4.6 (1.2)	0.076
HDL cholesterol, mmol/l*	1.2 (0.4)	1.2 (0.3)	1.2 (0.3)	1.3 (0.4)	<0.001
HbA_1c_ level, mmol/mol*	59.2 (19.9)	59.8 (20.1)	60.5 (20.8)	59.2 (18.9)	0.764
eGFR	72.4 (16.7)	71.9 (16.9)	73.8 (17.1)	71.5 (17.1)	0.237
Total number of medications prescribed^#^	3.8 (2.4)	4.0 (2.4)	4.0 (2.4)	3.8 (2.4)	0.088
Anti-hypertensive medication, n (%)	4497 (55.4)	3717 (58.4)	18773 (51.4)	5522 (59.7)	0.950
Lipid-lowering medication, n (%)	5512 (67.9)	4458 (70.0)	24918 (68.2)	6104 (66.0)	0.002
Hypoglycaemic medication, n(%)	5272 (65.0)	4337 (68.1)	26913 (73.6)	4563 (49.3)	<0.001

*Remission group determined for the 60,287 people who were alive at the first follow period (i.e. at 6 months following the start of the study) and therefore had follow-up data. Model F statistics from regression models reported here. Models used on imputed data were linear regression models for continuous variables, logistic regression models for binary variables, and ordered logistic regression models for ordered categorical variables (like IMD).

**Table 3:** Multinomial regression showing associations between weight change categories and group membership (compared to group 3).

	Unadjusted (n = 59743)				Adjusted^#^ (n = 59598)			
	Risk	95% CI		p-value	Risk	95% CI		p-value
	ratio				ratio			
% Weight change category								
**Group 1**								
No change or weight gain (from baseline) (n = 4360 (53.8%))	1.00				1.00			
Weight loss (≥2.5% to < 5%) (n = 610 (7.5%))	1.02	0.91	1.15	0.730	1.06	0.93	1.21	0.350
Weight loss (≥5 to <10%) (n = 815 (10.1%))	1.04	0.92	1.17	0.531	1.11	1.00	1.24	0.047
Weight loss (≥10%) (n = 2322 (28.7%))	0.99	0.92	1.07	0.769	1.17	1.08	1.26	0.000
**Group 2**								
No change or weight gain (from baseline) (n = 3573 (56.1%))	1.00				1.00			
Weight loss (≥2.5% to < 5%)(n = 470 (7.4%))	0.96	0.83	1.11	0.564	0.93	0.80	1.09	0.352
Weight loss (≥5 to <10%)(n = 608 (9.6%))	0.94	0.82	1.08	0.395	0.93	0.81	1.06	0.265
Weight loss (≥10%) (n = 1718 (27.0%))	0.89	0.81	0.98	0.017	0.89	0.81	0.98	0.017
**Group 4**								
No change or weight gain (from baseline) (n = 4700 (51.2%))	1.00				1.00			
Weight loss (≥2.5% to < 5%)(n = 658 (7.2%))	1.02	0.90	1.16	0.733	1.08	0.94	1.25	0.254
Weight loss (≥5 to <10%)(n = 954 (10.4%))	1.13	1.00	1.27	0.053	1.23	1.09	1.40	0.002
Weight loss (≥10%) (n = 2859 (31.2%))	1.13	1.06	1.21	0.000	1.42	1.31	1.54	0.000

^#^Adjusted model includes baseline weight, sociodemographic variables (age, sex, ethnicity and IMD), diabetes duration, number of co-morbidities and clustering within practices.

### Remission and CVD outcomes by remission trajectory

In our study cohort, 3,928 (6.5%) had a CVD event, 7,312 (12.1%) died, 4867 (8.1%) people had macrovascular complications, 15,527 (25.8%) had microvascular complications during the study period. In Cox models, people with type 2 diabetes who achieved remission at any point during the seven-year follow-up had a significantly lower risk of CVD events, macrovascular complications and microvascular complications, in both unadjusted and adjusted models. People with type 2 diabetes who achieved remission at any point during the seven-year follow-up had a significantly lower risk of all-cause mortality in adjusted models. These results are shown in S1 Table.

Group 3 (stable high HbA_1c_ levels) was assigned as the reference category for our Cox modelling. Compared to this group, people in all the remaining groups had a lower risk of developing microvascular complication in both unadjusted and adjusted models and those in Groups 1 and 4 also had lower risk of macrovascular complications and CVD events (in unadjusted and adjusted models). The risk of these complications was lowest for people in Group 4 who started off and remained at low HbA_1c_ levels (<48mmol/mol or <6.5%) for the entire seven-year follow-up period. Those in Group 1 (who had earlier decrease below HbA_1c_ <48mmol/mol or 6.5%, followed increasing HbA_1c_ levels) also had significantly lower risk of complications compared to Group 3. These results are shown in Table 4.

**Table 4:** Association between remission group and CVD outcomes in the CHIA type 2 diabetes cohort.

		Unadjusted model					Adjusted model*			
		HR	95% CI		p-value		HR	95% CI		p-value
**Macrovascular complications**	N = 48,942					N = 48,829				
Group 3 (ref)		1					1			
Group 1		0.85	0.78	0.93	<0.001		0.83	0.75	0.92	<0.001
Group 2		0.97	0.88	1.06	0.490		0.91	0.82	1.00	0.054
Group 4		0.70	0.64	0.77	<0.001		0.66	0.61	0.71	<0.001
**Microvascular complications**	N = 41,609					N = 41,527				
Group 3 (ref)		1					1			
Group 1		0.63	0.60	0.66	<0.001		0.65	0.61	0.70	<0.001
Group 2		0.78	0.74	0.82	<0.001		0.80	0.76	0.85	<0.001
Group 4		0.56	0.53	0.59	<0.001		0.59	0.55	0.64	<0.001
**CVD events**	N = 53,218					N = 53,097				
Group 3 (ref)		1					1			
Group 1		0.76	0.69	0.84	<0.001		0.74	0.67	0.83	<0.001
Group 2		0.94	0.85	1.04	0.254		0.88	0.79	0.98	0.021
Group 4		0.71	0.64	0.78	<0.001		0.67	0.61	0.73	<0.001
**Death**	N = 60,287					N = 60,138				
Group 3 (ref)		1					1			
Group 1		0.85	0.79	0.92	<0.001		0.82	0.76	0.89	<0.001
Group 2		1.01	0.93	1.09	0.829		0.92	0.85	1.01	0.086
Group 4		1.29	1.21	1.37	<0.001		1.11	1.03	1.19	0.004

Group 1 *achieving HbA*_*1c*_ *<48 mmol/mol (6*.*5%) followed by increasing HbA*_*1c*_ *levels*); Group 2 *decreasing HbA*_*1c*_ *levels*); Group 3 *stable high HbA*_*1c*_ *levels)*; Group 4 *stable low HbA*_*1c*_ *levels (<48mmol/mol or <6*.*5%)*. The adjusted model shown above included sociodemographic variables (age, sex, ethnicity, IMD), baseline weight, diabetes duration, number of co-morbidities and clustering within practices.

People with event of interest prior to the start of study were excluded from the analysis.

CVD events included a composite of myocardial infarction, amputation and stroke. Microvascular complications included a composite of peripheral neuropathy, retinopathy, and nephropathy. Macrovascular complications include a composite of stroke, MI, coronary heart disease peripheral arterial disease (PAD) and amputation. All-cause mortality was death from any cause.

For risk of all-cause mortality, the rate at which HbA_1c_ levels below 48mmol/mol or 6.5% was achieved was important. Group 2 (decreasing HbA_1c_ levels, though not achieving remission) had similar risk of all-cause mortality to Group 3 (stable high HbA_1c_ levels). Group 1 (achieving HbA_1c_ levels <48 mmol/mol or <6.5% followed by increasing HbA_1c_ levels) had lower risk of all-cause mortality than Group 3. However, Group 4 (i.e., those who started and remained with low HbA_1c_ levels throughout and limited variation) had higher risk of all-cause mortality.

## Discussion

### Main findings

In this population-based cohort of 60,287 people with type 2 diabetes, remission was common with 19% of people achieving remission at some point for at least 6 months. Achieving remission regardless of duration or pattern of HbA_1c_ level and remission status over time was associated with a lower risk of microvascular complications, macrovascular complications, and CVD events. However, the risk of these complications and mortality varied according to remission trajectories over time.

### Comparison with existing literature

To our knowledge, this is the first study to describe long-term patterns of different type 2 diabetes remission trajectories and their associations with CVD outcomes and all-cause mortality in a population-based cohort. No previous studies have utilised group-based trajectory modelling in this way and instead have examined remission as single whole cohorts [11,12]. Many also include only limited follow-up (<12 months) and therefore have not been able to report on the risk of microvascular complications, macrovascular complication or death [11]. The findings extend our previous findings by highlighting that lower risk of CVD outcomes is achieved regardless of duration of remission, though patients with consistently low HbA_1c_ levels have lowest risk of CVD outcomes. Consistent with the observational studies and in contrast to some of the trials of glucose-lowering drugs [18], we observed consistent trends in unadjusted and adjusted models between remission group and a lower incidence of both macrovascular and microvascular complications. Weight loss of ≥10% was an important predictor of remission trajectory, which is consistent with previous findings on the link between weight loss and remission [4,17].

## Possible explanation for our findings

Although a significant proportion of patients achieved weight loss of ≥10% across each group, Group 4 had the highest proportion of patients in this group (31.2%) suggesting that weight loss of ≥10% was more likely in this group of people who had lowest levels of HbA_1c_ at baseline. The proportion of patients achieving weight loss of ≥10% in Group 2 was lower than the proportion achieving weight loss ≥10% in Group 3 (stable high HbA_1c_ levels) which may be unexpected but may be partly due to differences in baseline characteristics such as BMI across the two groups (Table 2) as well as a slightly larger mean decrease in HbA_1c_ level for Group 3 compared to Group 2 (Fig 1); In terms of all-cause mortality, we found that the trajectory of remission was important. Patients in Group 1 (i.e., those achieving remission followed by increasing HbA_1c_ levels) had lower risk of mortality than those in Group 3 (stable high HbA_1c_ levels). This finding suggests that glycaemic control over a period of time may result in improved long-term health outcomes, even following subsequent increases in HbA_1c._ Moreover, Group 1 may have lower risk of mortality due to having consistently lower HbA_1c_ levels compared to Group 3. This reflects increasing risk of mortality at higher levels of HbA_1c_ and is in line with some studies that have reported a linear or J shaped relationship between HbA_1c_ and mortality [19]. Similarly, patients in Group 4 (stable low HbA_1c_ levels with limited variation) had higher risk of mortality. It is possible that people in Group 4 included people who are unwell with a high risk of mortality (such as those with cancer) and therefore could be more likely to lose weight and go into remission. This unintentional weight loss could not be distinguished from intentional weight loss and might be a plausible explanation for some of the observed variations by remission group. Further research is needed to explore which patients achieving remission are at higher risk of mortality. Further work is needed in larger and longer cohorts with more ethnic and socially diverse populations to develop targeted and personalised interventions according to remission group.

### Strengths and limitations

A strength of the study is our large population-based cohort of 60,287 people with type 2 diabetes across a wide geographic region of Southern England including 150 GP practices. The cohort included heterogeneity in age, sex and disease profiles but was limited to mainly people from white ethnicity. This reflects the local area but may not be generalisable to more diverse populations. We included a reasonable follow-up period of seven years with most previous studies examining remission limited to shorter durations [10]. The dataset used is from routinely collected clinical records and is dependent on clinicians accurately recording clinical events and thus is subject to error. However, we used Quality Outcome Framework measures wherever possible which are used for payment and administrative purposes. These measures have previously undergone validity testing and have high levels of completeness and accuracy [13]. We did not have exact dates for deaths in the database with only quarter of death available, so we used the mid-point of the quarter of death in the time to event analyses which may have introduced some error in our analysis. A further limitation was that we were only able to account for prescribed drugs that were captured in the electronic record; we did not have data on whether oral hypoglycaemic drugs were obtained from other sources or the exact date these drugs were prescribed in primary care. Missing data was another limitation of our study which is common with routinely collected data. Although, our sensitivity analysis demonstrated the robustness of our imputation methods as similar associations were observed in the non-imputed analysis. It is possible that some of our findings may be due to chance as we did conduct a number of hypothesis tests. Given that we observed consistent trends across all models and groups, this is less likely. Finally, we cannot rule out reverse causality.

## Conclusions

Remission of type 2 diabetes at any point during the course of diabetes is common in routine clinical care but patterns of remission including maintenance, vary considerably. People who achieve remission, even for shorter periods of time, continue to benefit from this lower exposure to hyperglycaemia, which may, in turn, lower the risk of CVD outcomes including mortality.

## Supporting information

S1 Table Association between remission and incidence of CVD outcomes and mortality over seven-year follow in the CHIA type 2 diabetes cohort. (DOCX)

## References

[1] Khan MAB, Hashim MJ, King JK, Govender RD, Mustafa H, Al Kaabi J. Epidemiology of type 1 diabetes- global burden of disease and forecasted trends. Journal of Epidemiology and Global Health. 2020; 10(1), 107–111. 10.2991/jegh.k.191028.001. 32175717 PMC7310804

[2] Steven S, Hollingsworth KG, Al-Mrabeh A, Avery L, Aribisala B, Caslake M, et al. Very low-calorie diet and 6 months of weight stability in type 2 diabetes: pathophysiological changes in responders and nonresponders. Diabetes Care. 2016;39:808–15. doi: 10.2337/dc15-1942 27002059

[3] Steven S, Taylor R. Restoring normoglycaemia by use of a very low calorie diet in long- and short-duration type 2 diabetes. Diabetic Medicine. 2015;32:1149–55. doi: 10.1111/dme.12722 25683066

[4] Dambha‐Miller H, Day AJ, Strelitz J, Irving G, Griffin SJ. Behaviour change, weight loss and remission of Type 2 diabetes: a community‐based prospective cohort study. Diabetic Medicine. 2020;37(4):681–688. doi: 10.1111/dme.14122 31479535 PMC7155116

[5] Holman RR, Paul SK, Bethel MA, Matthews DR, Neil HA. 10-year follow-up of intensive glucose control in type 2 diabetes. The New England Journal of Medicine. 2008;359(15):1577–89. doi: 10.1056/NEJMoa0806470 18784090

[6] Zhou JJ, Schwenke DC, Bahn G, Reaven P; VADT Investigators. Glycemic variation and cardiovascular risk in the veterans affairs diabetes trial. Diabetes Care. 2018;41(10):2187–2194. doi: 10.2337/dc18-0548 30082325 PMC6150432

[7] Giugliano D, Maiorino MI, Bellastella G, Chiodini P, Esposito K. Glycemic control, preexisting cardiovascular disease, and risk of major cardiovascular events in patients with type 2 diabetes mellitus: systematic review with meta-analysis of cardiovascular outcome trials and intensive glucose control trials. Journal of the American Heart Association. 2019;8(12):e012356. doi: 10.1161/JAHA.119.012356 31166153 PMC6645638

[8] ACCORD Study Group; Buse JB, Bigger JT, Byington RP, Cooper LS, Cushman WC, et al. Action to control cardiovascular risk in diabetes (ACCORD) trial: design and methods. American Journal of Cardiology. 2007;99(12A):21i–33i. doi: 10.1016/j.amjcard.2007.03.003 17599422

[9] Gregg EW, Chen H, Wagenknecht LE, Clark JM, Delahanty LM, Bantle J, et al. Association of an intensive lifestyle intervention with remission of type 2 diabetes. JAMA. 2012;308:2489. doi: 10.1001/jama.2012.67929 23288372 PMC4771522

[10] Lean ME, Leslie WS, Barnes AC, Brosnahan N, Thom G, McCombie L, et al. Primary care-led weight management for remission of type 2 diabetes (DiRECT): an open-label, cluster-randomised trial. Lancet. 2018;391(10120):541–551. doi: 10.1016/S0140-6736(17)33102-1 29221645

[11] Lean MEJ, Leslie WS, Barnes AC, Brosnahan N, Thom G, McCombie L, et al. Durability of a primary care-led weight-management intervention for remission of type 2 diabetes: 2-year results of the DiRECT open-label, cluster-randomised trial. Lancet Diabetes & Endocrinology. 2019;7(5):344–355. doi: 10.1016/S2213-8587(19)30068-3 30852132

[12] Hounkpatin H, Stuart B, Farmer A, Dambha-Miller H. Association of type 2 diabetes remission and risk of cardiovascular disease in pre-defined subgroups. Endocrinology, diabetes & metabolism. 2021; 4 *(*3*)*, [e00280]. doi: 10.1002/edm2.280 34277996 PMC8279611

[13] Khan NF, Harrison SE, Rose PW. Validity of diagnostic coding within the General Practice Research Database: A systematic review. British Journal of General Practice. 2010;60:199–206. doi: 10.3399/bjgp10X483562 20202356 PMC2828861

[14] Nagi D, Hambling C, Taylor R. Remission of type 2 diabetes: a position statement from the Association of British Clinical Diabetologists (ABCD) and the Primary Care Diabetes Society (PCDS). British Journal of Diabetes.2019;19:73–76.

[15] Hippisley-Cox J, Coupland C, Vinogradova Y, Robson J, Minhas R, Sheikh A, et al. Predicting cardiovascular risk in England and Wales: prospective derivation and validation of QRISK2. British Medical Journal. 2008;336(7659):1475–82. doi: 10.1136/bmj.39609.449676.25 18573856 PMC2440904

[16] Nagin DS, Odgers CL. Group-based trajectory modeling in clinical research. Annual Review of Clinical Psychology. 2010;6:109–38. doi: 10.1146/annurev.clinpsy.121208.131413 20192788

[17] Dambha-Miller H, Hounkpatin H, Stuart B, Farmer A. Associations between weight change and remission of type 2 diabetes: a retrospective cohort study in primary care. Practical Diabetes. 2021; 38(5), 8–14a. 10.1002/pdi.2355.

[18] Cryer PE. Death during intensive glycemic therapy of diabetes: Mechanisms and implications. The American Journal of Medicine. 2011;124:993–6. doi: 10.1016/j.amjmed.2011.08.008 22017775 PMC3464092

[19] Arnold LW, Wang Z. The HbA1c and all-cause mortality relationship in patients with type 2 diabetes is J-shaped: a meta-analysis of observational studies. Rev Diabet Stud. 2014 Summer;11(2):138–52. doi: 10.1900/RDS.2014.11.138 25396402 PMC4310064

