# Supplementary material for: Type 2 diabetes remission trajectories and variation in risk of diabetes complications: A population-based cohort study
Source: PLoS One. 2023 Aug 29;18(8):e0290791. doi: 10.1371/journal.pone.0290791 (PMC10464964; doi:10.1371/journal.pone.0290791)
Supplement: S1 Table — (DOCX) [file pone.0290791.s001.docx]

**Supplementary Table 1: Association between remission and incidence of CVD outcomes and mortality over seven-year follow in the CHIA type 2 diabetes cohort ***

|  |  | **Unadjusted** | | | |  | **Adjusted**  **^#^** | | | |
| --- | --- | --- | --- | --- | --- | --- | --- | --- | --- | --- |
|  |  | **HR** | **95% CI** | | **p-value** |  | **HR** | **95% CI** | | **p-value** |
| ***Macrovascular complications*** | *N=48942* |  |  |  |  | *N=48829* |  |  |  |  |
| Remission |  |  |  |  |  |  |  |  |  |  |
| No |  | 1 |  |  |  |  | 1 |  |  |  |
| Yes |  | 0.81 | 0.74 | 0.89 | <0.001 |  | 0.82 | 0.74 | 0.91 | <0.001 |
| ***Microvascular complications*** | *N= 41,609* |  |  |  |  | *N=41,527* |  |  |  |  |
| Remission |  |  |  |  |  |  |  |  |  |  |
| No |  | 1 |  |  |  |  | 1 |  |  |  |
| Yes |  | 0.54 | 0.51 | 0.58 | <0.001 |  | 0.57 | 0.54 | 0.61 | <0.001 |
| ***CVD events*** | *N=53,218* |  |  |  |  | *N=53,097* |  |  |  |  |
| Remission |  |  | |  |  |  |  |  |  |  |
| No |  | 1 |  |  |  |  | 1 |  |  |  |
| Yes |  | 0.79 | 0.69 | 0.80 | 0.001 |  | 0.80 | 0.70 | 0.92 | 0.002 |
| ***Death*** | *N=60,287* |  |  |  |  | *N=60,138* |  |  |  |  |
| Remission |  |  |  |  |  |  |  |  |  |  |
| No |  | 1 |  |  |  |  | 1 |  |  |  |
| Yes |  | 0.98 | 0.91 | 1.05 | 0.564 |  | 0.92 | 0.86 | 0.99 | 0.035 |

^#^Adjusted model includes baseline weight, sociodemographic variables (age, sex, ethnicity and IMD), diabetes duration, number of co-morbidities and clustering within practices. *People with event of interest prior to the start of study were excluded from the analysis.
